# Playing with Numbers: The Social and Behavioural Impacts of Using a Card Game to Teach Business Metrics

**DOI:** 10.3390/bs15060761

**Published:** 2025-06-01

**Authors:** Ruth Smith, Elaine Conway

**Affiliations:** 1 Accounting and Finance, University of Leicester School of Business, Leicester LE2 1RQ, UK; 2 Loughborough Business School, Loughborough University, Loughborough LE11 3TU, UK; e.a.conway@lboro.ac.uk

**Keywords:** Social Learning Theory (SLT), game-based learning, peer-to-peer learning, social interaction, skill development

## Abstract

This study investigated the social and behavioural impacts of employing a card game designed to support the teaching of business metrics through active peer-to-peer engagement, contrasting with traditional passive lectures. Grounded in Bandura’s Social Learning Theory (SLT), the study used a multiple-methods approach including student feedback, a focus group, and an interview. A novel card game, Metrics Masters©, was played by 390 students across a range of educational levels and settings. The research found that the game effectively introduced and reinforced their understanding of key business metrics, while simultaneously enhancing social interaction, teamwork, and problem-solving among Millennial and Generation Z students. The findings underline the efficacy of game-based learning and its close alignment with the SLT principles of observation, imitation, and social interaction. The theoretical contribution of this paper lies in its explicit application and extension of SLT within the context of business education, illustrating empirically how social interactions facilitated by game-based activities significantly enhance learning outcomes. Furthermore, this paper contributes to educational practice by providing robust evidence that game-based learning methods can effectively address educational challenges heightened by the COVID-19 pandemic, offering actionable strategies for educators aiming to foster both academic and social development among students.

## 1. Introduction

The current generation of university students are typically Millennials and Generation Z (Gen Z). Numerous studies have indicated that they take a different approach to learning compared with previous generations of students (Cilliers, 2017; Moncada & Moncada, 2014; Szilágyi et al., 2025). Evidence suggests that these generations have shorter attention spans and prefer active and visually stimulating learning environments (Hernandez-de-Menendez et al., 2020). This has implications for traditional, passive lecture-based teaching strategies, which may fail to engage current student cohorts effectively (Keller, 1987).

Teaching technical subjects, such as business metrics, may bring additional challenges. Topics such as the return on capital employed (ROCE) or environmental, social, and governance performance are often perceived as abstract or disconnected from real-world applications (Kolb, 1984; Prince, 2004), reducing motivation and engagement. This perception is further compounded by cognitive load: the technical complexity and volume of metrics can overwhelm students if not presented in structured, digestible formats (Sweller, 1988).

Social Learning Theory (SLT) (Bandura, 1977) posits that learning occurs within a social context, emphasising the role of observation, modelling, and imitation. This theory suggests a more active learning experience may engage and support deeper learning, which may appeal to the current generations of students more effectively, particularly in technical subjects. Despite numerous studies supporting SLT, its practical application within business education remains relatively limited.

This article investigated the social and behavioural impacts of employing a Top Trumps©-style card game designed for teaching business metrics through active peer-to-peer engagement, contrasting with traditional passive lectures. Grounded in Bandura’s (1977) SLT, the study used a multiple-methods approach including feedback, focus groups, and interviews. The research provides a unique contribution by demonstrating how the card game effectively introduces and reinforces students’ understanding of key business metrics while simultaneously enhancing social interaction, teamwork, problem-solving, and engagement among Millennial and Generation Z students.

This study was particularly timely, as the current cohorts of university students experienced considerable impacts on their educational journeys due to the COVID-19 pandemic. As many university courses moved online due to necessity during the pandemic, the importance of the social aspects of learning was lost (Sangster et al., 2020). This resulted in some students feeling isolated and struggling to maintain motivation and engagement with their studies and interaction with their peers within a learning environment (Freitas et al., 2020). This study found that a game-based approach can encourage social interaction and foster peer-to-peer learning through the creation of an engaging and hands-on experience, which resulted in the development of key employability and social skills.

These findings underline the efficacy of game-based learning and its close alignment with the SLT principles of observation, imitation, and social interaction. The theoretical contribution of this paper lies in its explicit application and extension of SLT within the context of business education, illustrating empirically how social interactions facilitated by game-based activities significantly enhance learning outcomes. Furthermore, this paper contributes to educational practice by providing robust evidence that game-based learning methods can effectively address educational challenges heightened by the COVID-19 pandemic, offering actionable strategies for educators aiming to foster both academic and social development among students.

## 2. Literature Review

Social Learning Theory (SLT) (Bandura, 1977) posits that learning occurs within a social context, emphasising the role of observation, modelling, and imitation. Previous studies have demonstrated the effectiveness of observational learning in education, suggesting that interactive, game-based environments significantly enhance students’ retention and understanding of complex concepts (Gee, 2003; Whitton, 2012). SLT (Bandura, 1977) is based on the idea of observational learning (modelling), where individuals observe and imitate the behaviours of influential or admired others, known as “models”. These can be parents, teachers, peers, or media figures. SLT emphasises four key mediational processes:

1. Attention (focusing on relevant behaviours);
2. Retention (remembering what was observed);
3. Reproduction (the ability to perform the observed behaviour);
4. Motivation (having a reason to imitate (such as expected rewards or avoiding punishment).

Self-efficacy is also a key element of Bandura’s (1977) SLT: this is the belief that an individual possesses the ability to meet situational demands or perform tasks. Bandura (1977) believed that social comparison information is a key component in the development of self-efficacy, which is a crucial factor determining students’ learning behaviours. Potosky (2002) found that self-efficacy is encouraged through playing games. Other studies have also found that using educational games is highly effective in developing self-efficacy in different subject areas (Meluso et al., 2012).

This idea of self-efficacy is also allied to Vygotsky’s theory of the Zone of Proximal Development (ZPD) (Vygotsky, 1978), which suggests that learning is most effective when students are supported in the space between what they can achieve independently and what they can accomplish with guidance from more knowledgeable peers or educators. According to Vygotsky, this support or “scaffolding” enables learners to progressively internalise new knowledge and skills, eventually mastering tasks they previously found challenging. The ZPD theory emphasises the crucial role of social interaction and collaboration in cognitive development, advocating for educational practices that actively engage learners within their optimal developmental zone. This is supported by ensuring that teaching materials and experiences are constructively aligned with learning outcomes (Biggs, 1996; Bruner, 1961).

Bloom’s Taxonomy (Bloom, 1956) is a widely recognised hierarchical framework designed to aid educators in structuring learning activities and assessment tasks progressively to address learning outcomes from basic knowledge recall to higher-order cognitive processes such as critical evaluation and original creation. By aligning educational interventions with Bloom’s Taxonomy, educators can systematically support students’ development from foundational comprehension to sophisticated, independent thinking and application (Biggs, 1996; Bruner, 1961). Building on this, Keller (1987) posited that learning occurs when students’ attention is captured, they see relevance in what they are learning (to their lives or future careers), they feel confident with their learning, and they experience satisfaction from the learning experience. This is the basis for the Attention, Relevance, Confidence, and Satisfaction (ARCS) methodology (Keller, 1987) and can also support educators in the development of teaching resources to achieve learning outcomes and address different learning styles and encourage deeper learning. Games are one example of how using the ARCS methodology could enhance potential teaching resources to support engagement and learning by appealing to a range of learners with different learning styles (Innes, 2015).

The use of games has captured human imagination and motivation for thousands of years (Chapman & Rich, 2018). Games can enhance thinking and unite individuals from diverse cultures and backgrounds as they have a universal language. Games can provide a set of boundaries to give experiences meaning; they allow players to explore and try things out in a “safe” environment (Kapp, 2012). Naik (2017) suggests that non-digital games increase social interaction and enhance communication between participants, plus they have the benefit of being able to be played anywhere. Vogel et al. (2006) highlighted that interactive games were more effective than traditional classroom instruction and aided cognitive skill development.

Innes (2015) highlights that good games can be engaging and challenging because there are variations in play and different outcomes every time the game is played. Moncada and Moncada (2014) mention that games should be easy to use and not too complex. Top Trumps© is one such game; it is simple to understand, easy to learn, enjoyable to play, and requires no prior knowledge (Abdullah Fadag, 2016). The participants can understand the rules quickly, and it can be easily customised for a variety of different topics. Winning the game depends on selecting the category that is “higher” or “lower” than that on your opponent’s card. Cohen (2014) notes that the use of games can transform dull lessons into more interesting, fun, and active experiences. Top Trumps©-style learning can be used to reinforce learning and interactions between students, with the benefit of it being customizable to any subject. However, Zagal et al. (2006) stress the importance of the players caring about the outcome of the game in order to be motivated by it.

The current students are typically Millennials (those born between 1981 and 1996) and Gen Z (those born from 1997 to the early 2010s) (Dimock, 2019). These generations have been the first to experience technology and access to the internet from an early age, which has fundamentally shaped their way of learning and thinking (Binabise et al., 2024; Szilágyi et al., 2025). Evidence suggests that these generations learn differently from previous generations. They have shorter attention spans and a preference for visually stimulating and active learning experiences (Cilliers, 2017; Hernandez-de-Menendez et al., 2020; Moncada & Moncada, 2014). Prince (2004, p. 223) defines active learning as “any instructional method that engages students in the learning process. In short, active learning requires students to do meaningful learning activities and think about what they are doing.” Park et al. (2019) also supported the idea that learning is more effective when students are active participants rather than passive bystanders. Board games, as interactive experiences, can be effective in promoting an active learning environment which appeals to young people, especially those from Gen Z (Sincharoenkul et al., 2020).

Many students have little real-life experience and knowledge (Mastilak, 2012). By participating in active learning through playing games, students can grasp concepts more quickly and make better connections between theory and practice (K. W. Braun & Drew Sellers, 2012; Pelser-Carstens & Seugnet Blignaut, 2018). To promote the development of real-life skills, Pelser-Carstens and Seugnet Blignaut (2018) suggest that universities should embed skills that are required within a workplace into teaching; the incorporation of board games can help with developing these skills as games simulate real-world activities and improve social relationships, including teamwork. Ilias et al. (2012) identified that the key skills developed from working in teams include leadership, coordination, decision-making, communication, adaptability, and interpersonal skills.

Salomon’s (1993) theory of distributed cognition emphasises that learning is not confined to the individual mind but is shared across people, tools, and environments. According to this perspective, cognitive processes can be distributed among the members of a group, between a person and a tool (such as a calculator or diagram), or across time through collaborative dialogue and shared memory. This framework shifts the focus from isolated learning to socially and contextually situated cognition, highlighting how meaning is co-constructed through interaction. In educational contexts, distributed cognition underscores the value of collaborative learning activities, where knowledge emerges from the interplay between learners and their environment, rather than from direct transmission alone. This makes it particularly relevant for examining interactive, game-based learning, where knowledge is built collectively through shared problem-solving and decision-making.

While there is literature supporting the use of game-based learning, both Tobias et al. (2011) and Boghian et al. (2019) discuss the potential challenges in isolating and measuring the actual learning effect from using games. De Freitas (2018) suggests that more evidence-based and scientific research is needed and warns that this may be a reason for some educators being reluctant to adopt a game-based approach. Molin (2017) includes busy teaching schedules and the fear of failure as barriers to adopting game-based learning.

Peer groups usually share a common ground, such as studying similar courses. A large part of students’ connection to their course is through engagement with their peers (Balakrishna, 2023). Mikropoulos and Natsis (2011) stated that students learn from their peers while playing a game together, something they would not experience if playing individually. Tanner and Lindquist (1998) reported that students enjoyed the social support from other team members and that playing a game improved the team spirit amongst players. Learning from peers through observation and enjoying social support are key factors many students missed out on during the COVID-19 pandemic (Sangster et al., 2020), hence why it is important to ensure that these experiences are incorporated where possible in the current university curriculum.

The social isolation from the COVID-19 pandemic not only restricted opportunities for peer learning, but there were also impacts for students due to increased stress and mental health challenges caused by online learning, social distancing, and wider anxiety issues (Liu et al., 2022). Social skills, in particular, which are key employability skills, are likely to have suffered as a result of social distancing, as they are behaviours which are acquired through interaction with one’s social environment by experiencing behavioural reinforcement, rule-based learning, and the modelling of acceptable behaviours (Freitas et al., 2020).

This study addressed the challenges of teaching business metrics to Millennial and Gen Z students, who experienced the COVID-19 pandemic during their educational journey. It achieved this by incorporating a novel interactive card game, based on Social Learning Theories, to appeal to their learning styles and improve engagement, social skills, and peer learning.

## 3. Methodology

The aim of this study was to assess whether a card game designed to teach a range of business metrics would support learning, in particular social learning. A multi-method approach was used by having students play the game and capturing participant feedback, but also through obtaining feedback from the staff involved in running the game through a staff focus group and interview. One or both authors were present at every session apart from one (where the interview was conducted afterwards) so were able to observe the game being played. This provided a rich dataset to assess the card game and its impacts on technical learning (the metrics) and also social learning.

Thematic analysis (V. Braun & Clarke, 2006) was used to derive the main themes emerging from both the student and staff data. Thematic analysis was chosen for this study as it offers a flexible yet robust approach to identifying, analysing, and interpreting patterns of meaning across qualitative data (V. Braun & Clarke, 2006). Unlike content analysis, which tends to focus on quantifying the frequency of codes or categories (Krippendorff, 2013), thematic analysis prioritises depth and contextual understanding, making it particularly suitable for exploring the nuances of participants’ experiences with social learning. While methodologies such as grounded theory (Charmaz, 2006) and the Gioia method (Gioia et al., 2013) are valuable for theory generation, they require iterative data collection, theoretical sampling, and a substantial volume of data, conditions that were beyond the scope of this study. Moreover, the emphasis of grounded theory on developing new theoretical frameworks contrasted with the goal of this study to extend an existing theory, Social Learning Theory, through empirical exploration. Thematic analysis, in contrast, enables a rich, detailed examination of learner reflections and collaborative dynamics, without the prescriptive demands of more rigid methodologies, making it particularly appropriate for research conducted in applied educational contexts.

**Figure 1:**
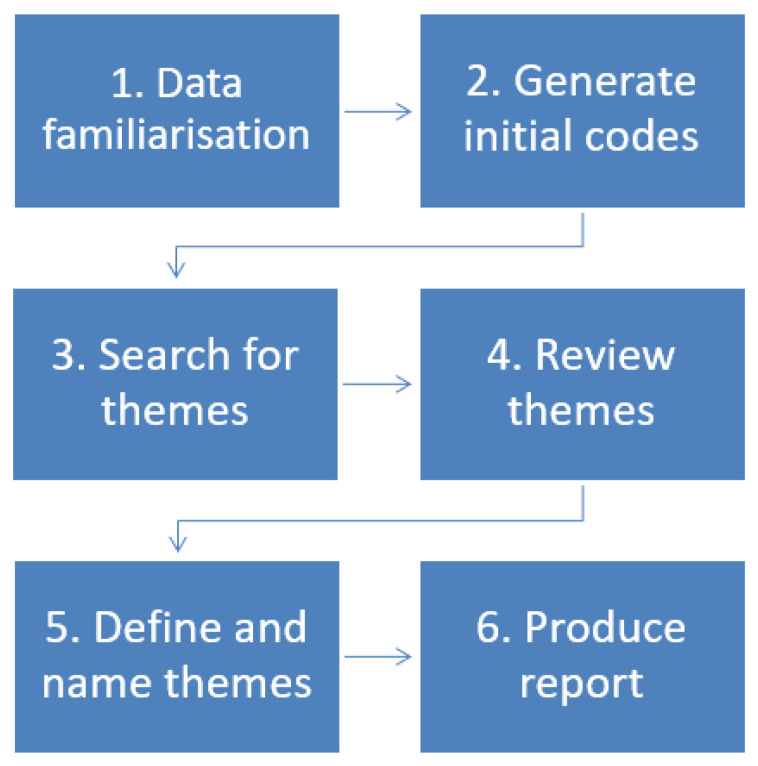
Phases of thematic analysis (V. Braun & Clarke, 2006).

Thematic analysis uses a six-phase approach, as shown in Figure 1 (familiarisation with the data, generating codes and initial themes, reviewing the themes, defining the themes, and writing up) as outlined by V. Braun and Clarke (2006). This was the process followed by this study, as discussed further in Section 3.4 below.

This study adopted an interpretivist paradigm, which supported an in-depth qualitative and inductive approach. Interpretivism argues that knowledge is subjective and based on people’s experiences and their understanding (Bryman & Bell, 2018). Saunders et al. (2009) state that interpretivism views the world as complex and made up of multiple realities, which are constantly changing. An inductive approach fits well with interpretivism as it is flexible in acknowledging that there can be multiple opinions. It allows for the creation of new theories and knowledge by identifying patterns and themes from the data collected (Saunders et al., 2009).

### 3.1. The Game: Metrics Masters©

**Figure 2:**
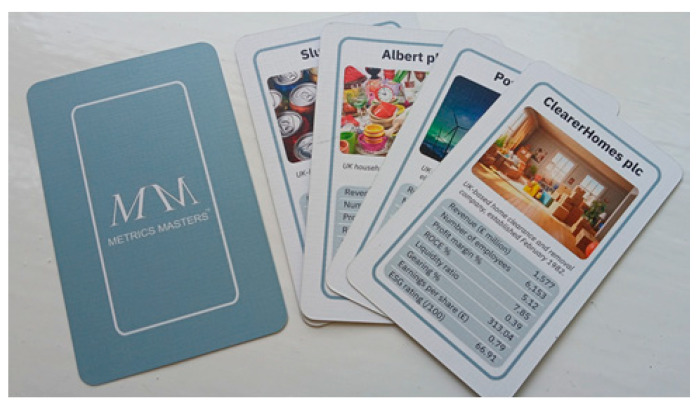
Examples of Metrics Masters^©^ cards.

A card game, called Metrics Masters©, was developed based on the popular game Top Trumps©, using real data from 160 anonymised companies from the London Financial Times Stock Exchange (FTSE) across a range of industries. Each company was depicted on a card, with a fictitious company name, an eye-catching picture to illustrate the company, brief information about the industry and the date of incorporation of the company, and a selection of eight metrics across a range of accounting and business measures, both financial and non-financial. Figure 2 shows examples of the cards.

The metrics were

- revenue;
- number of employees;
- profit margin;
- return on capital employed;
- liquidity;
- gearing;
- earnings per share;
- environmental, social, and governance (ESG) score.

The metrics were chosen through consultation with business school academics who engage in teaching metrics. A pilot version of the cards was trialled initially, and based on feedback from students and staff, the final choices were made. It was decided to incorporate both financial and non-financial metrics to encourage students to think more holistically about the issues facing companies. The financial metrics selected are commonly used in industry and reflect a range of income statement and balance sheet measures, but which also assess issues such as the availability of cash in the business to fulfil short- and long-term requirements. When analysing an organisation, a range of different metrics should be used to build up a complete picture: the metrics were chosen to reflect good practice in considering profitability, liquidity, gearing, and shareholder metrics (Bull, 2007). Given the breadth of the industries depicted on the cards, not all ratios, for example, inventory turnover, would have been appropriate, so the metrics were also chosen based on their broad applicability to all the industries used. The non-financial metrics (number of employees, date of incorporation, and ESG score) were chosen to provoke discussion about the issues around the firm size and complexity and whether newly established firms are more likely to perform well financially and to introduce concepts such as environmental activities, social and workplace policies, and governance issues that relate to how a company is managed. Given the increasing importance of ESG both within the investor community (Euronext, 2024) and as part of professional accounting body skillsets (ACCA, 2023), it was felt that it was also important to include this metric on the cards to broaden students’ appreciation of the wider issues facing businesses. Equally, by including the industry sector on each card, students could consider the impact of the industry on the different metrics when choosing a metric to play.

The game was developed based on Keller’s (1987) Attention, Relevance, Confidence, and Satisfaction (ARCS) framework for creating teaching materials which promote the four different facets which support effective learning. It was pre-tested in a pilot study using a small number of participants and revised based on class and tutor feedback prior to the main study.

### 3.2. The Process

The game was intended to support and reinforce the learning of business metrics, and hence it was used with students after a short micro-teach to present the metrics. In the micro-teach, each of the metrics was discussed, and it was agreed whether a “higher” or “lower” value would be preferable for each as the basis for the competitive element of the game. The rules of the game were introduced to those previously unfamiliar with the format. Students then self-organised into pairs to play against another pair. The game then commenced and was run with tutor support to clarify any queries for various periods of time. Different iterations of the game were played during the session, based on the time allowance and extent of the student discussions.

### 3.3. The Sample

The game was played by 390 students across different educational levels, ranging from UK qualification level 3 (the year prior to university, typically a student aged 18) to level 8 (PhD) (UK Government, 2025) as can be seen from Table 1.

The game was played on a variety of separate occasions, with the group size ranging from small groups (the minimum number playing was four participants) to a large induction event with one hundred students, as can be seen in Table 2. This assessed how well the game could be played in different physical settings.

In some settings, the participants were either not previously known to each other or did not know each other particularly well (for example, they may have been fellow students in a large class but had never had much personal interaction with each other previously). On the taster and applicant days and at the induction event, the participants had never met before. In the small classroom settings, the participants were at a mixture of educational levels as these were organised in a response to a general cross-campus call for participants, with a single exception where one friendship group of eight played together. There was a wide range of levels of previous exposure to business metrics in the sample, ranging from practically none to PhD business students.

Open-ended qualitative feedback was requested from the participants on a voluntary basis. The feedback was anonymous and no personally identifiable characteristics (gender, age, etc.) were collected in this study to encourage wider participation and due to the inherent challenges of using structured questionnaires at large events. However, all the students would have been classified as Millennials or Gen Z across the sample.

There were 144 individual feedback responses from the 390 participants (total response rate: 37%). The response rate was understandably lower at the larger events, but nonetheless, there was a wide variety of responses from participants at varying educational levels, as can be seen in Table 3.

In addition, a focus group with four staff members who facilitated some of the sessions was held, and an in-depth interview with another staff member who used the game with a large postgraduate class was conducted to gather a wider range of opinions and observations. The authors’ observations were used to inform the focus group and interview questions. The questions shown in Appendix A.1 and Appendix A.2 were structured around the game itself, the observations of the students during the sessions, the extent of social learning achieved from playing in pairs and small groups, the usefulness of the game in teaching business metrics, suggestions for improvements, and future applications in which the game could potentially be used. Both the focus group and interview were transcribed for analysis within NVivo software. The semi-structured questions used for the focus group and interview are included in Appendix A.1 and Appendix A.2, respectively.

### 3.4. Thematic Analysis Methodology

As stated earlier, thematic analysis (V. Braun & Clarke, 2006) was used to derive the main themes emerging from both the student and staff data. The six-phase approach (familiarisation with the data, generating codes and initial themes, reviewing the themes, defining the themes, and writing up) was used to analyse the qualitative data collected from written feedback, interviews, and focus groups. First, all the transcripts and written responses were read and re-read to ensure familiarisation with the content, during which initial notes were made. This was followed by the generation of initial codes across the dataset, supported by the use of NVivo software, identifying recurring features such as expressions of engagement, collaboration and reflections on the learning process.

These codes were then examined and grouped into potential themes that captured broader patterns of meaning, for example, “educational value” and “active engagement.” The themes were reviewed to ensure they accurately reflected the coded data and the dataset as a whole. They were then refined and clearly defined, with careful attention given to naming each theme in a way that communicated its core essence.

In the final phase, the themes were integrated into a coherent narrative that illustrated how participants experienced the card game activity and how these experiences aligned with and extended key elements of Social Learning Theory, such as modelling, observation, and reinforcement. This method allowed for a detailed, nuanced understanding of the social learning processes at play. Both authors independently coded the feedback and then compared the themes. These initial themes were then further classified into broader overall categories. The classification of sub-themes within the main five themes discussed below is presented in Appendix A.3.

To evaluate whether the game supported learning at different educational levels, the students’ feedback was also grouped by their educational level (levels 3–8) and analysed to determine whether there were any differences in perceptions or learning across the various levels. The findings from the thematic analysis and educational stage evaluation are discussed in the next section.

## 4. Findings and Discussion

### 4.1. Thematic Analysis

The key themes from the staff focus group, interview, and student feedback are discussed in this section. The examples have been labelled to indicate whether the feedback came from the staff focus group, interview, or students.

#### 4.1.1. Educational Value

The game helped participants understand financial metrics and their applications in a memorable way. It was particularly useful for beginners or those new to accounting and finance; for example, the feedback included the following statements: “I learned that for the gearing ratio, the lower the better” [student feedback] and “Learnt and consolidated understanding of ESG, Gearing ratio and business metrics” [student feedback]. This aligns with constructivist perspectives on active learning, preferred by Millennials and Generation Z, which emphasise the importance of engaging students in meaningful, context-based tasks (Biggs, 1996; Prince, 2004).

Even though the metrics were introduced during the micro-teach, there appeared to be a consolidation of learning during the game due to the replaying process: “I felt I learned more from the game than the teaching session” [student feedback]. Hence, the game effectively reinforced financial concepts, with some students finding it helpful for understanding and retaining knowledge; for example, one participant noted, “It recapped the ratios in a good way” [student feedback]. One student remarked, “Overall, an excellent way to learn. Much appreciated!” [student feedback].

Staff highlighted that the game “was a good thing to add… it’s easy access but adds something different to the flavour of teaching” [staff interview]. Another comment supported the game’s ability to engage students: “It brought it alive a little bit more. A topic that should be interesting… add an extra little tool to it” [staff interview] and “It’s very entertaining as well as educational” [staff interview].

They added that the game made the topic more accessible and enjoyable, providing a memorable and hands-on approach to learning and retaining concepts: “Recap helps with understanding” [focus group].

This feedback shows that the game has educational value by operationalizing Social Learning Theory in a way that deepens conceptual understanding through structured peer interaction. While Bandura (1977, 1986) primarily focused on behaviour acquisition through observation, this study extended his framework by showing how learners internalised abstract business metrics via repeated exposure to peers’ reasoning and decision-making. The act of replaying the game supported what Vygotsky (1978) describes as learning within the Zone of Proximal Development, allowing students to move from novice to more competent performance with peer scaffolding. Salomon’s (1993) concept of distributed cognition was also evident, as students engaged in meaning-making collectively rather than in isolation. Thus, the game fostered a learning environment where understanding was co-constructed, not just transmitted.

#### 4.1.2. Engagement and Enjoyment

The game was widely seen as a fun and engaging activity which encouraged interaction among students, even those initially hesitant to participate. It served as an effective icebreaker, fostering collaboration and communication in a social environment, particularly during events such as applicant or induction days, where students were unfamiliar with one another: “The game was fun, and it was a good way to interact with people I did not know” and “It is a very sociable game” [student feedback].

Participants enjoyed the interactive and social aspects, combining cooperation (working in groups or pairs) and competition (against other groups or pairs). The social aspects of the game were highlighted as a key feature, especially because many students missed out on formative experiences during the COVID-19 pandemic: “The game helped me to step out of my comfort zone and meet new people” [student feedback]. Another student responded, “Playing in teams is more motivating, because of the interaction with team members there is no pressure, so more enjoyable.” The competitive nature of the game added excitement and motivated participants to engage more fully; one student noted, “The game was fun and gave extra motivation to win” [student feedback]. This exemplifies Bandura’s (1977) mediational process of motivation: competition and the satisfaction of winning motivated students to learn and correctly apply business metrics, aligning with SLT’s idea that motivation drives imitation and learning. This shared experience often led to a stronger sense of camaraderie. As one participant observed, “Game was competitive and playful” [student feedback], while another reflected that “The satisfaction of winning was good” [student feedback].

The combination of competition and teamwork created a unique dynamic that strengthened relationships within the group and promoted the development of employability skills. The participants worked together to strategize, make decisions, and celebrate shared successes, further enhancing social bonds.

Breaking away from traditional learning methods and making often quite “dry” topics (such as business ratios and financial metrics) fun, the game helped to maintain student attention. For instance, one participant remarked, “It was really fun and a good way to get into ratios” [student feedback], while another noted, “I had a lot of fun playing the game, it helped me retain the metrics and how to measure the growth of a company” [student feedback]. This was also noted by staff: “For me, I would love to use it again next year… it helps add something different to teaching ratios that is often dull as dishwater” [staff interview].

Staff agreed on the ability of the game to engage students quickly: “After a few minutes there was a buzz in the room, and you could see that the students were enjoying the game” [focus group], “Loved seeing the student interaction” [focus group], and “there was plenty of noise in the room” [staff interview].

Engagement and enjoyment, often seen as affective outcomes, also play a critical cognitive role in social learning. Bandura (1986) highlighted motivation as a key element of observational learning; when learners are emotionally engaged, they are more likely to attend to and imitate behaviours. The game’s entertainment value, as noted in participant feedback, increased learners’ willingness to engage with complex content, enhancing their attentional processes and memory retention. This aligns with research by Gee (2003) and Whitton (2012), who argue that game-based environments naturally support intrinsic motivation, which in turn facilitates deeper learning. By embedding learning in an enjoyable social format, the game recontextualised Social Learning Theory within an affectively rich, learner-centred experience.

Additionally, the game illustrated SLT’s (Bandura, 1977) mediational processes in a variety of ways. For example, regarding attention, the game was engaging and attention-grabbing, critical for initiating this mediational process. The students were actively focused due to the game’s competitive and social nature. Regarding retention, many students explicitly stated that the game helped them remember the metrics better compared to traditional methods, highlighting how social interaction aids retention.

#### 4.1.3. Interactive and Collaborative Learning

Playing in teams was highlighted as being beneficial for developing social and employability skills, the acquisition of which many students missed out on during the COVID-19 pandemic. The game fostered communication, collaboration, brainstorming, problem-solving, and a sense of accomplishment. The hands-on, interactive nature of the game was seen as a refreshing way to reinforce concepts, improve retention, and motivate learners. It provided a platform for the participants to support one another and grow their confidence through teamwork in both social and academic contexts, as one student stated: “Fun way to interact with new people, very creative” [student feedback].

The participants benefitted from sharing perspectives and clarifying concepts together, particularly when individuals with various levels of expertise were paired, which deepened their understanding. By discussing the metrics with their peers, the participants gained the confidence to express their ideas and ask questions in a supportive environment. Students remarked, “Playing in teams helped grow my confidence” and “Playing in teams is good as it allows for discussion and reconsolidation on topics if unsure on them” [student feedback].

These findings aligned with the observational learning and modelling elements of SLT (Bandura, 1977). Through peer interactions, the mediational process of reproduction was demonstrated: students learned by observing how their peers chose metrics, discussed them with their team member(s), and made decisions. By observing their teammates’ reasoning, they internalised concepts (e.g., when gearing should be high or low), demonstrating observational learning. They then mimicked successful strategies used by their teammates or opponents, reinforcing the correct understanding of the metrics. They also demonstrated vicarious reinforcement (Bandura, 1977): by seeing the outcomes of decisions made by others, students learned about the metrics. Observing their peers succeed or fail when using certain metrics implicitly reinforced their understanding of which behaviours were beneficial.

Members of staff observed this dynamic, with one commenting, “Students were talking to each other easily and working together to achieve a common goal” [focus group]. Another stated, “This was a great example of creating social dimensions, helping students talk to each other and correct each other about the metrics” [focus group]. A student remarked, “It also motivated me to socialize once the game had started” [student feedback], emphasising the role of the game in creating a welcoming and interactive atmosphere.

The game effectively encouraged interaction among the participants, who may not already have known each other. This could be particularly valuable when used in diverse classrooms or during induction activities as a social icebreaker activity, encouraging teamwork and fostering new connections among the participants, particularly at induction-style events. It facilitated socialising and allowed the participants to meet new people, helping the participants step out of their comfort zones: “It is a great ice breaker with an inbuilt goal” [focus group]. For quieter or more reserved students, the game provided a structured way to engage socially. Staff noted this impact, stating, “Great to see students who were quiet at the start interacting with others” [focus group].

Interactive and collaborative learning lies at the heart of Social Learning Theory. This study reinforced and extended Bandura’s emphasis on learning through observation and imitation by showcasing how reciprocal teaching among peers, where students actively engage in questioning, explanation, and elaboration together, deepens understanding (Hall et al., 2007). The game fosters this reciprocal dynamic, turning passive learners into active co-constructors of knowledge, as learners move toward full participation in a learning community through interaction with more competent peers. These social exchanges are not incidental but central to how students internalise and apply financial concepts, offering a practical extension of SLT to complex, abstract domains.

#### 4.1.4. Practical Application

The game provided a real-world context, allowing students to connect theoretical concepts to practical applications. The participants appreciated its relevance to real-world contexts, particularly in understanding business metrics and industry comparisons. A participant noted, “Playing the game gives you a better feeling of which number in which category is high or low” [student feedback], while another appreciated seeing industry statistics, stating, “Interesting to see the statistics behind the companies we all know” [student feedback].

The game provided a practical way for students to apply theoretical knowledge, fostering a deeper understanding of financial metrics through active participation. The hands-on approach made the concepts more relatable and memorable: “It helped with my understanding of ESG, profit margins, and liquidity ratio” [student feedback]. The hands-on nature of the game encouraged active learning and knowledge construction, a concept posited by Constructivist Learning Theory (Bruner, 1961), which suggests that learners construct new knowledge through experiences. The feedback supported this: students said they “understood ratios better while playing” or “remembered more from the game than the lecture” [student feedback].

In addition, the game’s creative approach was appreciated as it stimulated interest in the subject. One pre-university student felt motivated to explore accountancy further after participating in the activity: “Makes me motivated to take accountancy” [student feedback].

Staff noted an increase in the students’ confidence in being able to apply their knowledge to real-world scenarios after playing the game: “By the end of it, they come out, ‘Oh, actually, you know, if I’m looking at a company now, I feel a bit more confident’” [focus group].

Social Learning Theory is often applied to observable behaviours, but this study extended the theory to the practical application of conceptual knowledge. Through gameplay, students not only understood business metrics but began to apply them in simulated business decisions. This bridged theory and practice in a social setting, aligning with Collins et al.’s (1989) model of cognitive apprenticeship, where learners acquire expertise through guided experiences of real-world tasks. Observing and discussing strategic decisions with peers mirrors the apprenticeship model and reinforces situated learning (Collins et al., 1989). The game thus demonstrates that social learning is not limited to behaviour modelling but can be adapted to foster the application of cognitive skills in contextually rich environments.

#### 4.1.5. Flexibility and Adaptability

The game is adaptable to different educational levels, making it versatile for use in various teaching settings. Cohen (2014) states that one benefit of using a Top Trumps-style game is that it is easily customisable to any subject. The participants also recognised this by suggesting its potential for broader use, with one remarking, “The topic and content could be changed and adapted to match any age group” [student feedback].

The game’s design was simple and intuitive, making it easy for students to grasp and participate without difficulty, which would be useful with classes of diverse cultural and educational backgrounds and varying levels of expertise to increase accessibility and enable all students to participate and contribute. The physical cards were highlighted as being particularly effective for promoting interaction during sessions. The student comments included “The physical factor (holding the cards, exchanging them, etc.) are more immersive than playing it online” and “Online might make it lose part of its essence” [student feedback].

Due to its simplicity and adaptability, staff noted that it was suitable for repeated use and integration into various teaching contexts, adaptable to different teaching goals: “It could be used several times” [focus group] and “Would be good to use as a starter in a lesson and ask students to make their own cards” [focus group].

Equally, staff felt that the game was adaptable to various group sizes and contexts, working well in both small tutorial groups and larger lecture-style settings, as it was typically played in small groups irrespective of the size of the class: “Pairs or small groups seem to work best… it is simple to understand, a great game” [focus group]. All the staff commented that they would use the game again in the future for promoting social learning within new groups and for teaching business metrics.

Hence, the game’s flexibility and adaptability demonstrate that social learning environments can be designed to accommodate diverse learner needs and contexts. Bandura (2001) later expanded his theory to include the notions of self-efficacy and agentic learning, through which individuals actively shape their learning trajectories. The ability to modify the rules, adjust the pace, and replay scenarios allows students to engage in personalised and adaptive learning within a social framework. This supports later interpretations of social learning that integrate learner autonomy with collaborative dynamics (Schunk & Zimmerman, 2007). The game’s adaptable design ensures that social learning is not a fixed process but one that evolves with learners’ goals, experiences, and social interactions.

### 4.2. Thematic Analysis to Extend Social Learning Theory Through Game-Based Learning

**Figure 3:**
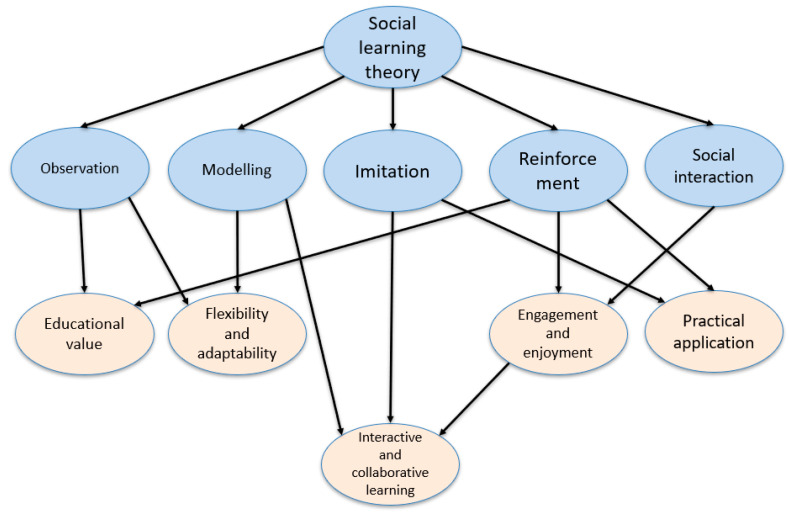
Schematic for the extension of Social Learning Theory based on the thematic findings.

These thematic findings extend SLT by demonstrating how a structured, game-based learning environment can operationalise and extend the theory’s core principles within a higher education context. At its foundation, SLT posits that learning occurs through observation, imitation, modelling, and reinforcement within a social context (Bandura, 1977) (shaded blue in Figure 3). The findings from our thematic analysis affirm these mechanisms while also revealing additional pedagogical dimensions made possible using an interactive card game (shaded light orange in Figure 3).

First, the theme of interactive and collaborative learning reflects the importance of modelling and social interaction in SLT, as students engaged in peer discussions, shared strategies, and corrected each other’s misunderstandings in real time. This peer-led dialogue not only facilitated imitation and reinforcement but also positioned learners as active participants in co-constructing knowledge, an aspect often underemphasized in traditional interpretations of SLT.

Second, the theme of engagement and enjoyment highlights the motivational role of positive reinforcement, demonstrating that fun and competitive elements can drive participation and deepen learning. The game’s ability to foster sustained attention and intrinsic motivation strengthens SLT’s emphasis on reinforcement, suggesting that affective engagement is a crucial, complementary factor in observational learning.

Third, the theme of practical application extends SLT by bridging theory with real-world relevance. Learners used financial metrics in authentic, contextualised scenarios, reinforcing the idea that meaningful learning occurs when abstract concepts are applied socially and experientially. This positions the game as a form of situated modelling, where learners imitate financial reasoning in a safe, practice-oriented setting.

Additionally, the themes of educational value and flexibility and adaptability reveal how SLT can be expanded to accommodate diverse learner needs and subjects through adaptable, gamified pedagogies. These elements promote inclusive, scalable approaches to peer learning, especially when teaching complex or abstract content such as financial ratios.

Together, the five themes based on the findings of educational value, engagement and enjoyment, interactive and collaborative learning, practical application, and flexibility and adaptability demonstrate how Social Learning Theory can be extended beyond its traditional focus on behavioural modelling to encompass deeper cognitive, emotional, and contextual dimensions of learning. By embedding business metric education in a socially interactive, game-based environment, this study shows that learners not only observe and imitate but also co-construct knowledge, apply concepts in context, and adapt their learning through peer engagement and reflection. These findings build on Bandura’s foundational work by integrating principles from constructivism, distributed cognition, and cognitive apprenticeship, resulting in a more dynamic and participatory model of social learning suited to contemporary educational contexts.

These extensions are shown in Figure 3 to show that SLT is not only a descriptive model of social learning but also a foundation that can be actively integrated into pedagogical experiences. By incorporating game mechanics, team-based learning, and student autonomy, this research extends SLT into a more dynamic, application-oriented framework for contemporary education.

### 4.3. Areas for Improvement

Students and staff were also invited to offer suggestions for improvement, either to the game itself or to suggest ways in which it could be used more effectively. The most popular themes that emerged from this were as follows.

#### 4.3.1. Repetition and Lack of Variety

One criticism was the potentially repetitive nature of the gameplay, which diminished students’ engagement over time. One student noted, “The game was enjoyable, but it got more repetitive after a while” [student feedback]. Participants highlighted the limited number of metrics as a factor contributing to this issue. This can be mitigated by keeping the game short to maintain engagement and introducing additional metrics or scenarios to reduce repetition and encouraging discussion around the different industries. This could potentially include designing versions of the game tailored for different group sizes and levels of expertise, including more challenging versions for advanced learners.

#### 4.3.2. Lack of Deeper Understanding

While the game facilitated metric learning and recognition, it had limitations in promoting deeper learning and application. The students often focused on identifying whether the metrics were high or low without understanding the underlying calculations or significance. One participant expressed this limitation, saying, “Focuses more on retaining metrics but less on understanding how they are calculated” [student feedback]. This could be improved by incorporating more discussions or ratio calculations during or after the gameplay to deepen understanding.

Additionally, as the cards only contained a single year’s worth of data, there was a lack of comparative data (e.g., growth rates or sector averages), which restricted deeper analysis and learning opportunities, but this could be mitigated by providing more information about industry trends.

#### 4.3.3. Contextual Gaps

A few participants felt that some metrics lacked a clear context, reducing the game’s effectiveness. For instance, some participants would have preferred to know the real names of the companies and industries to make the metrics more meaningful: “It would have been useful to add growth rates or comparisons to sector averages to provide more context” [student feedback]. Equally, some students would have preferred to assess their cards’ significance with comparisons with benchmarks or examples: “Could give examples on what good and bad look like” [student feedback]. Staff from the focus group suggested the following improvement: “Could give the top 3 and bottom 3 ratio results for all categories so the students know where their card ranks” [focus group].

#### 4.3.4. Initial Uncertainty

Some students and staff noted challenges in understanding the gameplay initially, requiring additional guidance: “We did play the game wrong at the start, but I still learned about different companies” [student feedback], “They were unsure initially and some pairs needed a little prompting” [focus group], and “Some students needed more time to understand the mechanics before starting the game” [focus group]. Staff suggested a solution, stating, “A demonstration video on how to play the game would be good” [staff interview]. A short video would have the additional benefit of appealing to the learning style of today’s students.

#### 4.3.5. Balancing Gameplay

The mechanics of the game occasionally led to gameplay imbalances. While team-based play was a significant strength, it could also result in uneven participation. Larger teams sometimes led to situations where one or two members dominated, leaving others less involved. A participant noted this issue, stating, “In larger teams, it felt like only one person was playing” [student feedback]. Smaller groups or pair-based gameplay might better ensure that all participants are actively engaged. This would also make discussions more focused and inclusive.

In larger sessions, the opportunities for meaningful interaction between teams were reduced. The tiered or lecture-style setup of some classrooms could further hinder communication. A staff member reflected on this limitation, noting that while there was discussion and interaction within the teams, the broader classroom dynamics were less conducive to cross-team engagement. Encouraging discussions at key points during the game, such as between rounds, could strengthen social connections. Teaching staff could ask teams to reflect on their strategies or share insights with the broader group, fostering a sense of collective learning.

Dominant metrics (students repeatedly playing the same ones) could affect the gameplay dynamics. It was suggested that this could be improved by introducing new features, like multiple metric selections, ranking cards, declaring metrics, or allowing participants to create their own cards for increased customisation, or varying game formats (individual/team-based). One student’s suggestion was “Declare the metrics then throw the cards in the centre would make it more fun” [student feedback].

### 4.4. Themes and Learning by Educational Level

Analysing the feedback in the context of the individuals’ educational level to assess the themes, tone and cognitive depth of the game, the results showed that there was a clear progression of cognitive development, more active learning approaches, and critical thinking across the different levels, as summarised in Table 4 and discussed below.

As an example of the emphases identified in the student feedback, Appendix A.4 shows thematic clouds for the students’ feedback by their educational level. Whilst this shows the main themes at the basic word level, below is a more detailed analysis by students’ educational levels.

#### 4.4.1. Level 3 (Pre-University)

Students expressed their enjoyment of the game as the most common theme, repeatedly mentioning words such as “fun” and “engaging”, demonstrating a very positive tone. The game was also effective as an entry point into business education as respondents mentioned understanding terms like gearing ratio, ESG, or liquidity and which metrics should be high or low. However, this learning was likely only at a surface level at this stage. The respondents also appreciated the social interaction involved in the game, particularly as an icebreaker to get to know others.

#### 4.4.2. Level 4 (Undergraduate Year 1)

The feedback from this group indicated that they appreciated the game for supporting teamworking; they found it easy to play but wanted a slightly more complex understanding of the ratios. They also started to critique the gameplay mechanics (e.g., repetition, duration) and suggested improvements relating to the cards and metrics, showing more understanding of instructional design and increasing cognitive awareness.

#### 4.4.3. Level 5 (Undergraduate Year 2)

Students at this level began to reflect on the application of knowledge, with more critical thinking and reflection about the educational purpose of the game. For example, some respondents were starting to question how well the game helped them apply the ratios, not just memorise them. They also wanted to gain more contextual understanding from the cards, signalling a shift from rote understanding to deeper learning; for example, they wanted the real names of the companies on the cards and more information about the impact of the industry on the metrics and how they are used. They also showed a strong appreciation for team-based interaction to support their learning.

#### 4.4.4. Level 6 (Undergraduate Year 3)

The students provided critical insights into the pedagogical design of the game. They requested more analytical depth and suggested ways to increase cognitive engagement, such as industry-specific comparisons, in-game calculation tasks, and reducing the reliance on the “easy” metrics. They more critically evaluated how and why they were learning, showing an interest in the teaching mechanics and deeper cognitive engagement.

Some students also requested reminders of the calculations for the metrics as they had forgotten how some were calculated. This was possibly because a large number of the level 6 students in some of the sessions had returned to university after an industrial placement year and may not have had continued exposure to business metrics during that time.

There was also an interesting difference in opinions on the game format between level 6 students, notably regarding whether it should remain as a physical card game or whether it would be better in a digital format. The students were equally split between enjoying the tactile learning provided by the physical game and suggesting a digital version for speed or efficiency.

#### 4.4.5. Level 7 (Postgraduate)

Advanced learners critiqued the lack of conceptual reinforcement, suggesting the removal of oversimplified metrics or restructuring the gameplay to apply greater learning pressure (e.g., forcing certain metrics to be played at certain times). They also placed more emphasis on wanting to know why a certain ratio mattered, not just if it was high or low. Some also wanted to take the exercise further by using case studies and real-life scenarios to extend their learning. Hence, the feedback from this group reflected pedagogical awareness and an emphasis on strategic learning design and analysis.

#### 4.4.6. Level 8 (PhD/Doctoral)

Doctoral-level feedback focused on metacognitive learning. Students proposed gameplay mechanics that reinforced conceptual understanding, like getting students to devise cards for themselves and encouraging manual ratio calculations. They also suggested mixing individual/team modes and having different rounds, along with wanting more information about real-life data and more complex decisions that the ratios would apply to. As most of the PhD/doctoral students in the sample also teach undergraduate seminars, their suggestions reflected a dual perspective as learners and potential educators.

These findings align with other educational theories, such as Bloom’s Taxonomy (Bloom, 1956), which describes six levels of cognitive development, remembering, understanding, applying, analysing, evaluating, and creating, as students progress from knowledge recall (level 3) to evaluation and creation (levels 7–8). For instance, examples of the student feedback from each of the educational levels demonstrate the stages of the taxonomy, as shown in Table 5. The findings are consistent with those of Syed Ahmad and Hussin (2017), who found that the use of educational games promoted pedagogic learning and increased educational value with the application of Bloom’s Taxonomy. Challa et al. (2024) also found that the learning attributes of Generation Z students showed a positive relationship with the lowest and highest levels when applying Bloom’s Taxonomy in their study.

There was clear progression across the different levels of the taxonomy, with the comments at each level typically being repetitive, such as “it was fun” and “easy to play” at earlier levels, followed by a greater focus on the ratios and metrics comprehension, and transitioning to a greater emphasis on group interaction or learning mechanics at the higher levels, as highlighted in the thematic clouds in Appendix A.4.

There were only a few outliers; for example, a level 4 student used unusual language (possibly as English was not their first language): “The game was really fantasmagical and very interesting”. Additionally, a level 5 student demonstrated more analytical and evaluative thinking than is typical at their educational level: “I question how useful the game is. They have to understand certain topic meaning (definitions), but I feel as though there could be a way to apply that knowledge further.”

The findings also contribute to Vygotsky’s theory of the Zone of Proximal Development (ZPD) (Vygotsky, 1978). The game scaffolded learning effectively within students’ ZPD, especially at early levels. As Vygotsky emphasised, the ZPD is the space between what a learner can do alone and what they can do with help; at the lower levels (levels 3–4), the game acted within the ZPD, allowing students to learn with minimal guidance through interaction. At the next levels (levels 5–6), students began to express that they were prepared for deeper learning; in this case, they wanted more context regarding the companies on the cards and to apply their learning through real-world analysis. At the higher levels (levels 7–8), the students were reaching mastery of their subject and wanted to restructure the game itself by making improvements in how it could be used. This indicates that they had moved beyond the ZPD for this version of the game. Equally, as learners mature, they seek to refine or challenge the structure of the game, indicating higher-order constructivist activity (Bruner, 1961).

## 5. Conclusions

This study demonstrated the application and effectiveness of SLT, particularly in the context of using educational games to enhance learning through observation, imitation, interaction, and social engagement. Student and staff feedback indicated that the Metrics Masters© card game successfully engaged students, improved their understanding of business metrics, and encouraged social learning (Deci & Ryan, 1985; Vygotsky, 1978). Observational learning and peer modelling could significantly enhance educational outcomes. Team interactions allowed the students to observe and imitate effective strategies, directly linking to the increased retention and reproduction of knowledge (Bandura, 1986). The game’s competitive structure motivated students to actively participate and engage cognitively, aligning with SLT’s motivational aspect (Schunk & DiBenedetto, 2020). The feedback showed the interplay between the environment (classroom, game design), personal cognitive factors (students’ confidence and existing knowledge), and behaviour (interaction, decision-making). This aligns with SLT’s reciprocal determinism concept, highlighting that learning occurs through mutual interactions between these factors.

SLT emphasises that learning is inherently social. The social aspects of the Metrics Masters© game are among its most impactful features, fostering collaboration, breaking down barriers, and building confidence among participants. By optimising the team sizes, introducing structured social interactions, and providing clearer instructions at the start, the game’s role as a powerful tool for both social and educational development can be further enhanced. As the participants connected over shared challenges and successes, the game not only taught business metrics but also created a vibrant and supportive learning community, facilitating peer teaching and discussion. The students corrected and supported each other, reinforcing the idea that social contexts significantly enhance learning outcomes.

The game also positively influenced students’ motivation and confidence, with the competitive element and teamwork required providing a sense of engagement and accomplishment. However, the participants offered thoughtful suggestions to deepen its educational value. These included expanding the range and complexity of the metrics, incorporating more detailed industry contexts, and offering clearer explanations of the metric definitions and calculations. These suggestions reflect the desire of the students and staff to make the game more accessible and engaging. To further enhance the learning outcomes, the recommendations included integrating instructional resources and expanding the gameplay mechanics. Overall, the feedback underscores the game’s strong potential as a dynamic, flexible, and impactful learning tool in business education.

This study also demonstrated the adaptability and effectiveness of game-based learning across educational stages. As students mature academically, they demand more meaningful, context-rich, and challenging learning experiences. The card game functions as a scaffolded, constructivist tool that supports cognitive development (Bloom, 1956; Bruner, 1961; Vygotsky, 1978). As students move up the educational levels, their feedback reflects a transition from passive knowledge reception to critical evaluation and instructional insight, which shows learning theory in action. The card game provides a flexible platform that can be tailored to meet diverse learning objectives and developmental needs.

This research contributes to the theory by extending SLT into the under-explored area of game-based learning within business school higher education, offering empirical validation that structured, interactive games significantly enhance learning outcomes. It underscores that learning is inherently a social activity and demonstrates explicitly how educational games can leverage social contexts to foster deeper conceptual understanding, peer collaboration, and motivation.

From a practical perspective, this study also provides educators with actionable insights, demonstrating that game-based methodologies can effectively mitigate the educational and social challenges heightened by the COVID-19 pandemic, such as reduced confidence, diminished peer interactions, and student isolation. The adaptability and flexibility of the Metrics Masters© game enable educators to tailor its application to different educational levels, enhancing its relevance and impact. This could be extended to other academic disciplines to compare subject-appropriate metrics across different applications, for example, in medical, geography and similar practically focused subjects. In addition, the game could be adapted for a digital platform to appeal to other active learning styles of Millennials and Gen Z, which could also accommodate hybrid learning contexts.

This study has limitations in that it focused specifically on business education, using a single educational card game (Metrics Masters©). The results may not be directly transferable or generalizable to other educational disciplines or game formats without further empirical investigation. The effectiveness of the game-based intervention was evaluated primarily through immediate feedback, focus groups, an interview, and observations. Due to the nature of the events (for example, open days), it was impractical to conduct questionnaires, which would have contributed additional quantitative data to the study. Future research should address this to test the extended Social Learning Theory which the current qualitative results suggest. Equally, the absence of longitudinal data means the study did not assess the longer-term retention of knowledge or sustained behavioural and social changes over time.

Areas for future research include exploring the applicability and effectiveness of similar game-based interventions across different educational contexts and subjects beyond business education. It is also suggested that additional longitudinal and quantitative studies are conducted to assess the long-term effectiveness of game-based learning to investigate the sustained impacts of using educational games on knowledge retention, behavioural changes, employability, and social skill development over an extended period.

## Figures and Tables

**Table 1:** Game participants by educational level.

Qualification Level	Education Level	No. of Students
3	Pre-university year	123
4	First-year undergraduate	104
5	Second-year undergraduate	19
6	Third-year undergraduate	79
7	Masters’ degree/postgraduate	52
8	PhD/doctorate	13
Total		390

**Table 2:** Variety of settings and participants’ educational levels.

Game Setting	No. of Participants	Academic Level
Classroom (normal teaching session)	Two sessions (one with fifty, one with thirty)	One at level 7, one at level 6
Classroom (informal small groups)	Eight sessions with varying numbers of participants	Varying number at levels 4–8 in each session
University taster day	23 participants	Level 3
Applicant day	Two groups of twenty and two groups of thirty	Level 3
Induction event	100 participants	Level 4

**Table 3:** Responses by educational level.

Educational Level	Number of Respondents	Response Rate
3	17	14%
4	42	40%
5	19	100%
6	44	56%
7	9	17%
8	13	100%
Total	144	37%

**Table 4:** Evolution of cognitive development across educational levels.

Educational Level	Focus	Learning Approach	Depth
Level 3	Fun and social; basic concept retention	Passive reception	Surface-level
Level 4	Light application of critical thinking, basic suggestions	Slightly active	Emerging awareness
Level 5	Conceptual reinforcement, desire for context	Active interaction	Deeper concern with application
Level 6	Contextual analysis, learning mechanics	Critical engagement	Strategic thinking
Level 7	Strategic design, purposeful selection	Pedagogical reflection	High cognitive engagement
Level 8	Instructional improvement, applied learning design	Reflective and evaluative	Deep and metacognitive

**Table 5:** Bloom’s (1956) Taxonomy applied to the students’ feedback by their educational level.

Bloom’s Taxonomy Levels	Examples from Student Feedback	Metacognitive Developments	Education Level
Remember	“I learned what gearing ratio means”	Students are grasping foundational concepts.	Levels 3–4
Understand	“I now understand why lower gearing is better”	They start to apply knowledge.	Levels 4–5
Apply	“We used the metric in decisions during game”	Engagement with team discussion shows application and some analysis.	Levels 5–6
Analyse	“We should compare across industries”	Deeper critiques indicate analysis: suggestions show evaluative thinking.	Levels 6–7
Evaluate	“Game needs more context to be truly useful”	Learners assess the educational depth and request richer analytical contexts.	Levels 7–8
Create	“Let us make our own cards/cases”	They want to create knowledge, showing creativity and evaluative learning.	Level 8

## Data Availability

Restrictions apply to the dataset: The dataset presented in this article is not readily available because the data are part of an ongoing study. Requests to access the datasets should be directed to the corresponding author.

## Appendix A

### Appendix A.1. Focus Group Questions

- What did you think about the microteach?
- Any improvements you can suggest for the microteach?
- What did you observe about the students playing the game?
- What do you think about the game?
- What worked well in terms of the cards?
- What worked well in terms of the game play/dynamics?
- What could be improved in terms of the cards?
- What could be improved in terms of the game play/dynamics?
- Any other comments?

### Appendix A.2. Questions for Semi-Structured Interview

- What is the demographic of your students (e.g., level, previous experience of metrics)?
- Did you use the microteach materials, if so, what were your impressions?
- What would you change with the microteach (if used)?
- Any improvements you can suggest for the microteach?
- What did you observe about the students playing the game?
- What do you think about the game?
- What worked well in terms of the cards?
- What worked well in terms of the game play/dynamics?
- What could be improved in terms of the cards?
- What could be improved in terms of the game play/dynamics?
- Any other comments?
